# Bioorthogonal Radiolabeling of Azide-Modified Bacteria Using [^18^F]FB-sulfo-DBCO

**DOI:** 10.1021/acs.bioconjchem.4c00024

**Published:** 2024-03-14

**Authors:** Aryn A. Alanizi, Alexandre M. Sorlin, Matthew F. L. Parker, Marina López-Álvarez, Hecong Qin, Sang Hee Lee, Joseph Blecha, Oren S. Rosenberg, Joanne Engel, Michael A. Ohliger, Robert R. Flavell, David M. Wilson

**Affiliations:** † Department of Radiology and Biomedical Imaging, University of California, San Francisco, San Francisco, California 94158, United States; ‡ Department of Psychiatry, Renaissance School of Medicine at Stony Brook University, Stony Brook, New York 11794, United States; § Department of Medicine, University of California, San Francisco, San Francisco, California 94158, United States; ∥ Department of Radiology, Zuckerberg San Francisco General Hospital, San Francisco, California 94110, United States

## Abstract

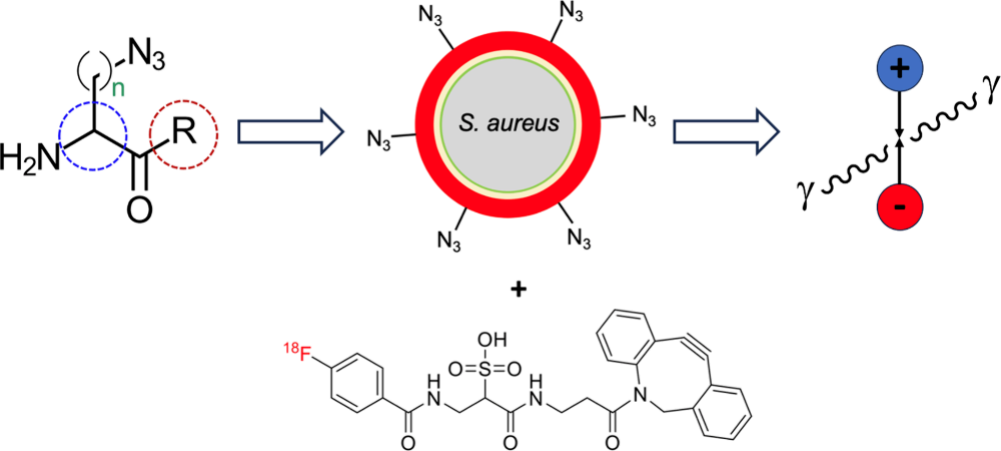

*Purpose:* This study was motivated by the need for better positron emission tomography (PET)-compatible tools to image bacterial infection. Our previous efforts have targeted bacteria-specific metabolism via assimilation of carbon-11 labeled d-amino acids into the bacterial cell wall. Since the chemical determinants of this incorporation are not fully understood, we sought a high-throughput method to label d-amino acid derived structures with fluorine-18. Our strategy employed a chemical biology approach, whereby an azide (-N_3_) bearing d-amino acid is incorporated into peptidoglycan muropeptides, with subsequent “click” cycloaddition with an ^18^F-labeled strained cyclooctyne partner. *Procedures:* A water-soluble, ^18^F-labeled and dibenzocyclooctyne (DBCO)-derived radiotracer ([^18^F]FB-sulfo-DBCO) was synthesized. This tracer was incubated with pathogenic bacteria treated with azide-bearing d-amino acids, and incorporated ^18^F was determined via gamma counting. *In vitro* uptake in bacteria previously treated with azide-modified d-amino acids was compared to that in cultures treated with amino acid controls. The biodistribution of [^18^F]FB-sulfo-DBCO was studied in a cohort of healthy mice with implications for future *in vivo* imaging. *Results:* The new strain-promoted azide–alkyne cycloaddition (SPAAC) radiotracer [^18^F]FB-sulfo-DBCO was synthesized with high radiochemical yield and purity via *N*-succinimidyl 4-[^18^F]fluorobenzoate ([^18^F]SFB). Accumulation of [^18^F]FB-sulfo-DBCO was significantly higher in several bacteria treated with azide-modified d-amino acids than in controls; for example, we observed 7 times greater [^18^F]FB-sulfo-DBCO ligation in *Staphylococcus aureus* cultures incubated with 3-azido-d-alanine versus those incubated with d-alanine. *Conclusions:* The SPAAC radiotracer [^18^F]FB-sulfo-DBCO was validated *in vitro* via metabolic labeling of azide-bearing peptidoglycan muropeptides. d-Amino acid-derived PET radiotracers may be more efficiently screened via [^18^F]FB-sulfo-DBCO modification.

## Introduction

The diagnosis and management of bacterial infection in clinical practice frequently rely on imaging, including plain films (X-rays), computed tomography (CT), and magnetic resonance (MR). These tools primarily evaluate the structural changes that accompany infection, including the presence of abnormal tissue or fluid collections. The nuclear medicine techniques positron emission tomography (PET) and single photon emission tomography (SPECT) may also be applied, for example, indium-111 white blood cell scanning using a patient’s own white blood cells to detect abnormal leukocyte accumulation.^1,2^ A major limitation of these methods is that they primarily detect the host immune response rather than the bacteria themselves, motivating the more recent development of PET radiotracers targeting bacteria-specific metabolism. This class of tracers uses metabolic pathways and structures that only bacteria possess, for example, the folate biosynthesis pathway and the special ability of bacteria to metabolize certain sugars and sugar alcohols including maltose, sorbitol, and mannitol. Following the groundbreaking report of Weinstein et al. validating the sorbitol derivative 2-deoxy-2-[^18^F]fluoro-d-glucose ([^18^F]FDS) as an Enterobacteriaceae-specific PET tracer,^3^ many tracers were subsequently explored including folate-targeted probes ([^11^C]trimethoprim,^4^ α-[^11^C]PABA,^5^ 2-[^18^F]F-PABA^6,7^) and sugars/sugar alcohols in addition to [^18^F]FDS including [^18^F] maltose derivatives^8−10^ and 2-[^18^F]fluoro-d-mannitol^11^ ([^18^F]FMtl). These radiotracers have been studied in numerous animal models of infection and more recently in patients with promising results.^12,13^

Our laboratory is particularly interested in musculoskeletal infections including vertebral discitis-osteomyelitis (VDO),^14^ its typical nontuberculous causative pathogen *Staphylococcus aureus*, and PET radiotracers targeting the bacterial cell wall.^15^ Based on a report describing bacterial incorporation of exogenous d-methionine, we initially developed d-methyl-[^11^C]-methionine as a PET tracer using a trivial modification of its common l-enantiomer radiosynthesis via [^11^C]methyl iodide,^16^ later developing an in-loop method using an acyclic homocysteine precursor^17^ and studying the tracer in patients with suspected prosthetic joint infections.^18^ We also developed a radiosynthesis of d-[3-^11^C]alanine, which showed remarkable sensitivity to bacterial infections in animal models of myositis, pneumonia, and VDO.^19^ For these carbon-11 tracers, the major limitation is the short half-life (*t*_1/2_ = 20 min), making it potentially difficult to produce tracers on-demand in acute care/inpatient settings. In addition, this short half-life requires an on-site cyclotron, which would restrict access to these tools, which can only be generated in major academic centers. We have therefore focused on developing *S. aureus*-sensitive radiotracers using the longer *t*_1/2_ radionuclide fluorine-18. Numerous reports in the fluorescence literature have indicated promiscuous incorporation of modified d-amino acids in bacterial peptidoglycan, allowing facile detection of the cell wall and elucidation of microbial biology.^20−23^ These studies have suggested that metabolic labeling of the bacterial cell wall using fluorine-18 is likely feasible, which motivates the current approach.

**Figure 1:**
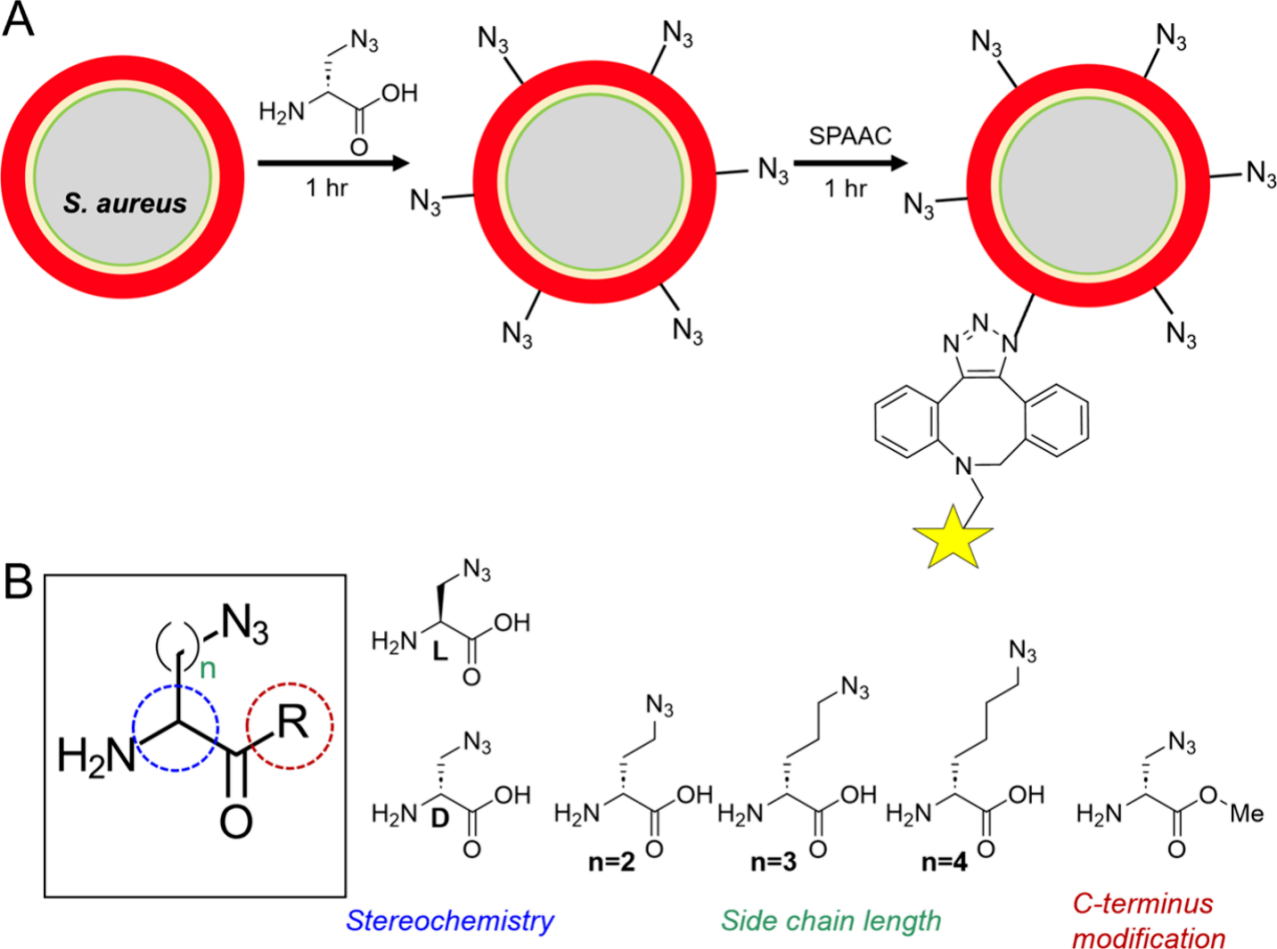
**Strain-promoted azide–alkyne cycloaddition (SPAAC) strategy for metabolic labeling of*S. aureus*.** (A) Analogous to Bertozzi’s work, an azide-bearing artificial d-amino acid is incorporated into the muropeptides of *S. aureus* peptidoglycan and subsequently labeled with a labeled strained cyclooctyne for detection. In this work the cyclooctyne was modified with a dye for fluorescent/visual detection or with ^18^F via [^18^F]SFB-derived [^18^F]FB-sulfo-DBCO. (B) Potential modifications of the amino acid scaffold for the metabolic labeling of pathogenic bacteria. We considered using D vs L amino acids (2-position stereochemistry), side-chain length (1, 2, 3, and 4 added methylene groups), and modification of the C terminus via a methyl ester control. Modification of the C-terminus has been shown to have a profound effect on the incorporation of exogenous d-amino acids with both decreased and increased uptake vs the carboxylate parent.

However, despite extensive and compelling data in the literature, the structural determinants of unnatural d-amino acid incorporation are not fully understood. For fluorine-18 tracer development, the serial design and radiosynthesis of various d-amino acid-derived PET tracers are remarkably costly and inefficient. In developing these radiopharmaceuticals, we sought a method whereby aspects of the d-amino acid scaffold could be more rapidly explored, including variable side-chain structure/length and C-terminal modification, as suggested by a recent report indicating both positive and negative effects of carboxylate substitution on fluorescent d-amino acid accumulation.^24^ Motivated by previous work in the Bertozzi lab,^25,26^ we hypothesized that this search might be facilitated using a bioorthogonal chemistry approach, whereby an exogenous d-amino acid derived bearing a side-chain azide is incorporated into bacterial peptidoglycan and subsequently “discovered” via cycloaddition using a detectable strained cyclooctyne^27^ (Figure 1). Analogous methods have targeted other components of the bacterial cell wall including its *N*-acetyl muramic acid^28,29^ and 3-deoxy-d-manno-2-octulosonic acid (KDO)^30,31^ content, using bioorthogonal chemistry. For both fluorescence and PET, these cell-wall specific structures may be used to visualize living bacteria.^15^ In this work, we developed a water-soluble and fluorine-18 labeled strained cyclooctyne prosthetic group ([^18^F]FB-sulfo-DBCO) analogous to previously reported fluorescent dibenzocyclooctyne derivatives that could be used to generate pathogen-specific PET signals. We believe that this strategy could be used to establish a “pre-targeting” infection imaging method for which a patient-delivered d-amino acid would be followed by a PET tracer used to detect it. Understanding that clinical applications might require a bioorthogonal imaging approach using faster cycloaddition chemistry,^32^ we chose strain-promoted azide–alkyne click chemistry (SPAAC) ligation for our proof-of-concept study based on the fluorescence literature.^25^ Using a d-amino acid probe with a small side-chain (i.e., d-azido-alanine) would likely facilitate bacterial incorporation due to resemblance to d-alanine itself, whose robust bacterial incorporation is well-established.^33^ Fura et al. showed an inverse relationship between side-chain size and bacterial accumulation for d-amino acid-derived fluorescent probes,^20^ and our previous work demonstrated that d-[3-^11^C]alanine incorporation was significantly higher than that of d-[methyl-^11^C]methionine by bacteria *in vitro*.^17,19^ This concept would also have the advantage of sensing only peptidoglycan-incorporated d-amino acids in contrast to signals generated using d-[3-^11^C]alanine, for example, which may be incorporated into bacteria and mammalian cells via other mechanisms. To date, most pretargeting approaches for PET have targeted cancer^34−36^ and dementia^37^ with numerous elegant studies published in the past decade. A recent report describing the detection of *S. aureus* using a radiolabeled antibody suggested the potential value of pretargeting,^38^ partially motivating the current study.

In this paper, we describe the successful detection of azide-modified peptidoglycan in *S. aureus* using [^18^F]FB-sulfo-DBCO and highlight its use in exploring modified d-amino acid incorporation. In the future, this approach may be used to detect living bacteria *in vivo*, which may require more reactive bioorthogonal cycloaddition pairs.

## Results

### Fluorescence Detection of N_3_-Modified Bacteria Using Strain-Promoted Azide–Alkyne Cycloaddition

**Figure 2:**
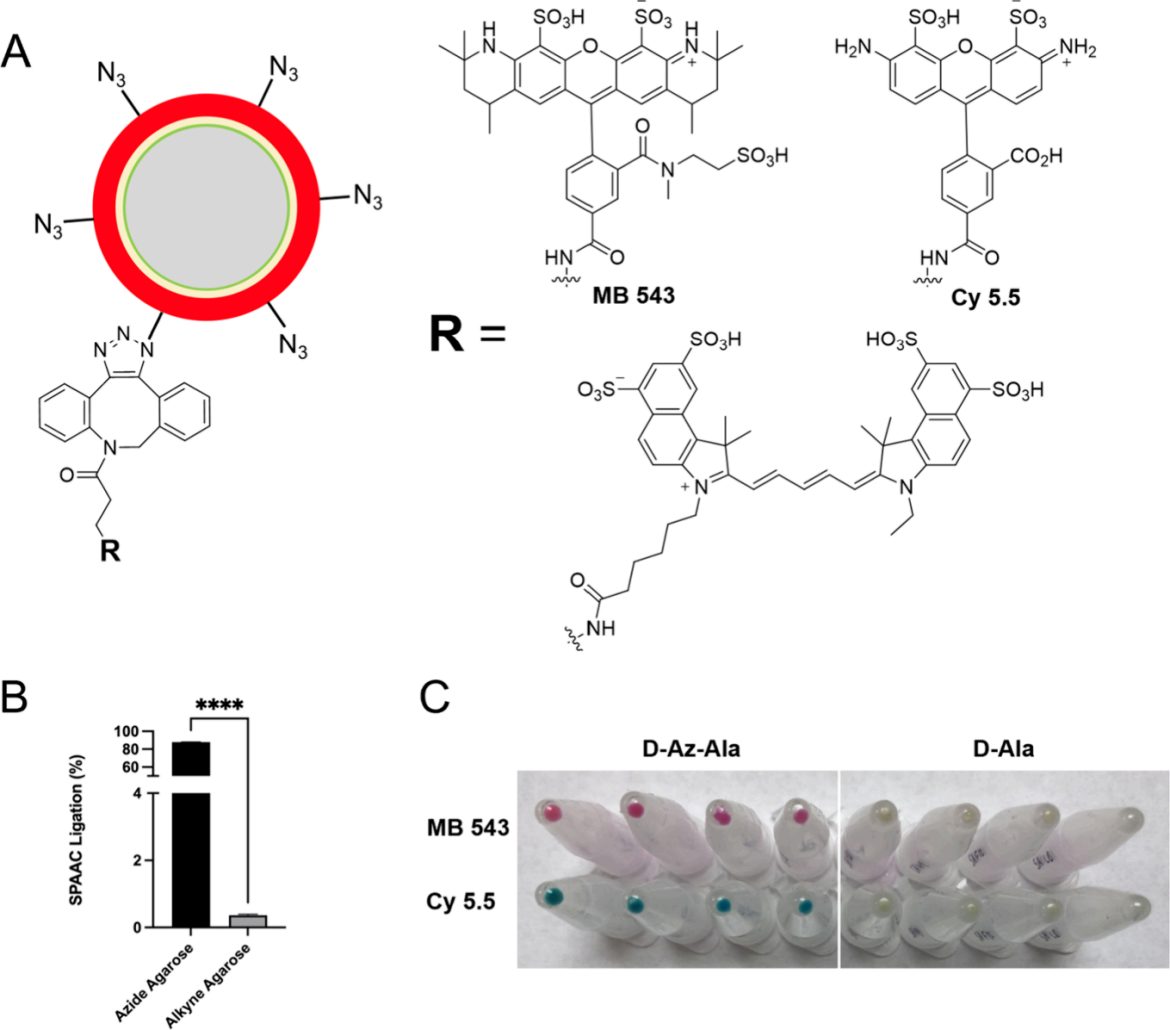
**Fluorescence/visual detection of N**_**3**_**-modified bacteria using a reported strained cyclooctyne method.** (A) Chemical structures of dibenzocyclooctyne (DBCO) conjugates of MB 543 (543 nm λ_ex_, 566 nm λ_em_), Cy 5.5 (683 nm λ_ex_, 703 nm λ_em_), and AF 488 (488 nm λ_ex_, 496 nm λ_em_). The first two dye conjugates were chosen for easy visual identification of muropeptide-retained signal, while AF 488 was employed for improved quantification of fluorescent signals. (B) Quantification of signals generated by AF 488 ligation to azide-labeled agarose beads was measured to test the feasibility of SPAAC ligation. Agarose beads were separated from media to measure SPAAC fluorescence. Azide agarose was used as a positive control to approximate the upper limit of measurable fluorescence, while alkyne agarose was used as a negative control. Data are represented as a percentage of fluorescent SPAAC ligation to the agarose beads in comparison to its filtrate (*N* = 3). (C) A series of SPAAC reactions in *S. aureus* grown (left to right) in PBS, M9, F-12, and LB. d-Azido-alanine can be visualized via ligation with the alkyne-labeled fluorophore partners MB 543 (DBCO) and Cy 5.5 (DBCO). In contrast, the negative control d-Ala does not react with fluorophores bearing alkynes.

In preparation for PET studies, we initially developed an efficient workflow to screen azide-modified bacteria using fluorescent dyes, including MB 543, Cyanine 5.5, and Alexa Fluor 488 (Figure 2A, Supporting Information Figure S1) analogous to previously reported cycloadditions for unnatural amino acid-containing muropeptide detection. Before attempting fluorescent modification of bacteria, we first tested AF 488 (DBCO) SPAAC ligation using commercially available azide- and alkyne-modified agarose beads. As expected, AF 488 (DBCO) resulted in lower signals when incubated with the alkyne agarose compared to the agarose-azide control (*P* < 0.001) (Figure 2B). We also investigated the chemical stability of d-azido-alanine and showed using chiral stationary phase HPLC absence of racemization in mouse and human sera over 3 h at 37 °C (Supporting Information Figure S3). We subsequently incubated *S. aureus* with d-azido-alanine in several media: Phosphate Buffered Saline (PBS), M9 minimal Media (M9), Ham’s F-12 (F-12), and Lysogeny Broth (LB) for 1 h. Following serial centrifugation and washing, the bacterial pellet was resuspended in media with MB 543 (DBCO) and Cy 5.5 (DBCO) for SPAAC ligation with facile visualization of ligation (Figure 2C) for all media tested. In contrast to the retained color for d-azido-alanine-treated bacteria, there was minimal SPAAC seen for d-alanine-treated *S. aureus* controls, a visual result further quantified for AF 488 (DBCO) ligation (Supporting Information Figure S3). These results formed the basis for subsequent studies using the cyclooctyne PET tracer [^18^F]FB-sulfo-DBCO.

### Radiosynthesis of [^18^F]FB-sulfo-DBCO

**Figure 3:**
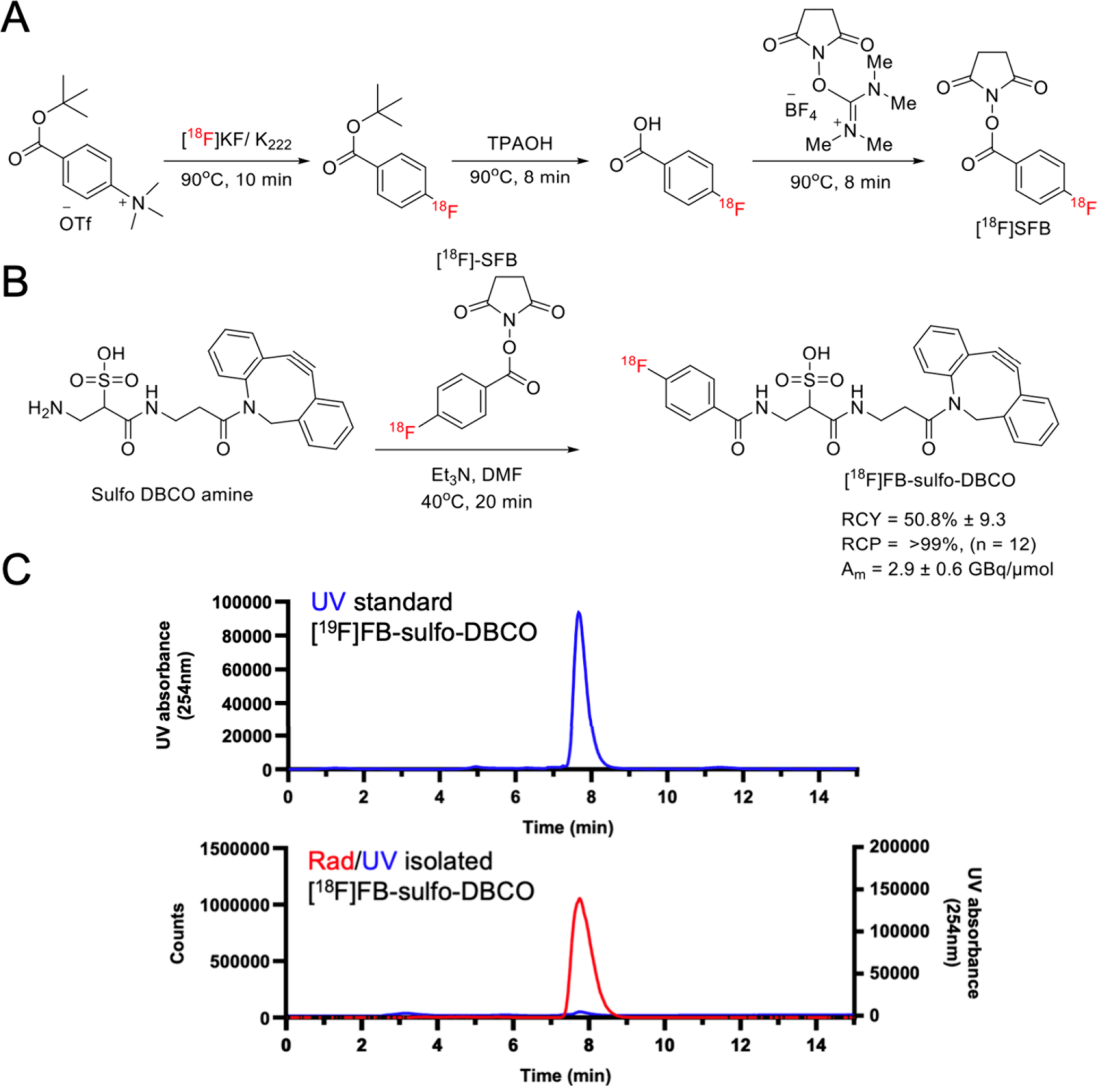
**Radiosynthesis and characterization of [**^**18**^**F]FB-sulfo-DBCO.** (A) Synthesis of [^18^F]SFB. (B) Synthesis of [^18^F]FB-sulfo-DBCO (*N* = 12). The radiosynthesis of [^18^F]FB-sulfo-DBCO was accomplished via [^18^F]SFB using a commercially available cyclooctyne precursor and purified via preparative HPLC. (C) Radiochemical analysis of [^18^F]FB-sulfo-DBCO. Analytical HPLC was used to confirm the chemical identity of the radiotracer via coelution with a characterized nonlabeled standard.

Based on initial concerns that a hydrophobic cyclooctyne-derived PET probe might result in increased nonspecific binding, we sought a water-soluble PET tracer compatible for SPAAC ligation with robust and reproducible radiosynthesis. Based on the commercial availability of the strained cyclooctyne-containing and sulfated amine Sulfo DBCO amine, we envisioned rapid amide formation via the readily available activated ester *N*-succinimidyl 4-[^18^F]fluorobenzoate ([^18^F]SFB)^39^ (Figure 3A), analogous to other reported fluorine-18 prosthetic groups.^40,41^ The amine precursor was reacted with [^18^F]SFB for 20 min at 40 °C in the presence of Et_3_N. The reaction mixture was purified via semiprep HPLC using a Phenomenex Luna C18 column. The calculated radiochemical yield (RCY) for [^18^F]FB-sulfo-DBCO starting from [^18^F]fluoride was 50.8% ± 9.3 (decay corrected) with radiochemical purity of >99% and a calculated molar activity of 2.9 ± 0.6 GBq/μmol (Figure 3B). Analytical HPLC was used to confirm the identity of the new radiopharmaceutical [^18^F]FB-sulfo-DBCO versus an NMR/HRMS-characterized cold standard (Figure 3C).

### PET Labeling of Pathogenic Bacteria Including *S. aureus* Using [^18^F]FB-sulfo-DBCO

**Figure 4:**
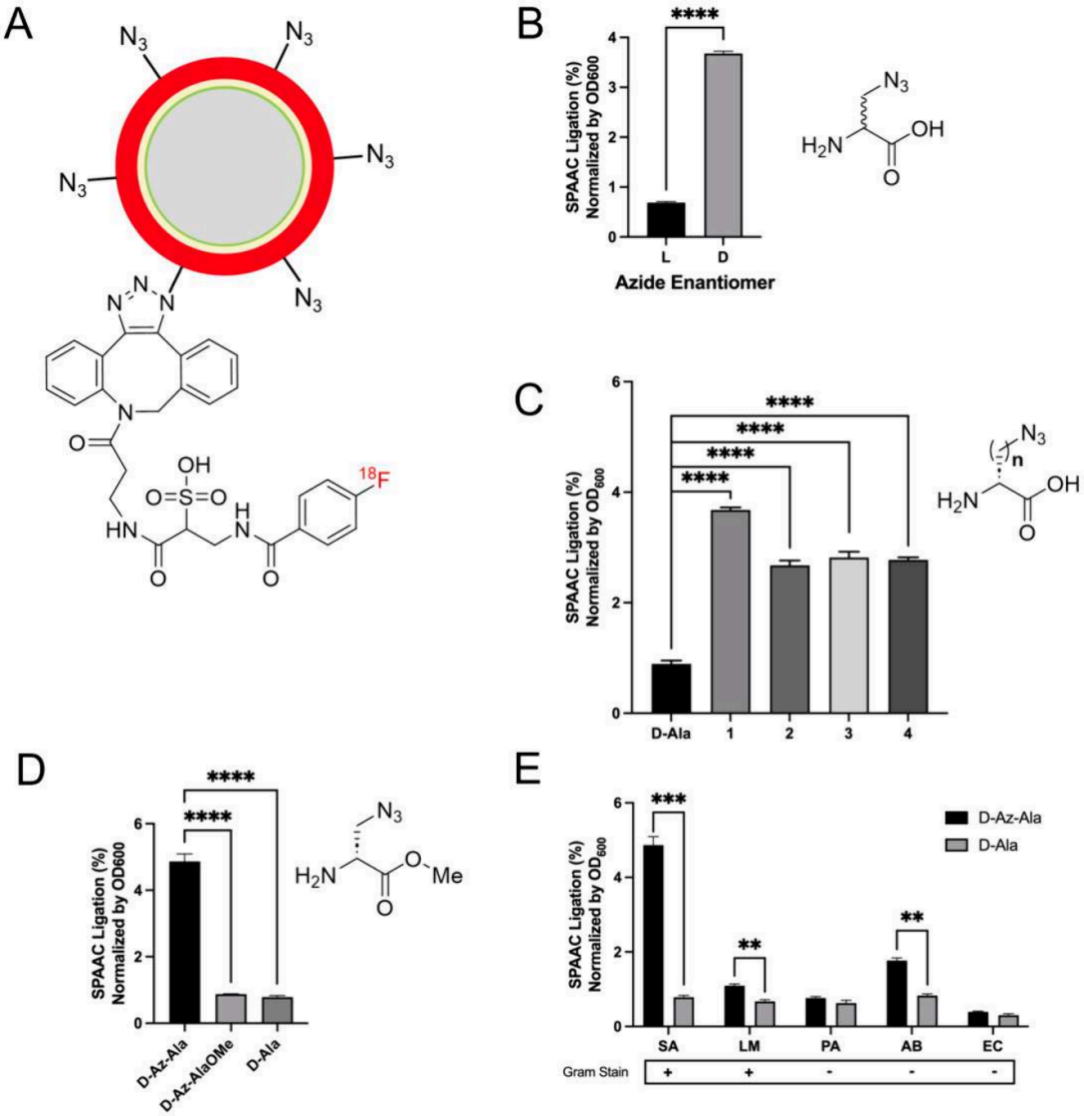
**SPAAC radiolabeling of pathogenic bacteria using [**^**18**^**F]FB-sulfo-DBCO.** In all cases, incorporated radioactivity was determined using a gamma counter. All data are represented as a percentage of radiolabeled SPAAC ligation to the bacterial pellets in comparison to the filtrate, normalized by their final measured OD_600_ (*N* = 3 per experiment). (A) Cycloaddition product of d-azido-alanine-modified *S. aureus* and [^18^F]FB-sulfo-DBCO. (B) SPAAC ligation using d-azido-alanine vs l-azido-alanine modified *S. aureus*, showing higher uptake for the d-amino acid N_3_ partner (5.3 fold, *N* = 3, *p* < 0.000001). (C) The effect of increased N_3_d-amino acid side chain length via increasing the number of methylene additions (1–4) on SPAAC incorporation in *S. aureus*. The highest labeling was observed for d-azido-alanine (1). (D) As an additional control, ligation using d-azido-alanine-treated *S. aureus* was compared to that using its corresponding methyl ester d-azido-alanine-OMe; this modification has been reported to decrease the incorporation of exogenous d-amino acid-derived structures. The SPAAC ligation using d-azido-alanine-treated *S. aureus* resulted in higher signal (5.6 fold, *N* = 3, *P* = 0.000061) vs methyl ester treatment and higher signal (6.2 fold, *N* = 3, *P* = 0.00006) vs treatment with d-alanine itself. (E) Using the d-azido-alanine [^18^F]FB-sulfo-DBCO method to label a panel of clinically significant Gram-positive and Gram-negative pathogens (SA, *S. aureus*; LM, *L. monocytogenes*; PA. *P. aeruginosa*; AB, *A. baumannii*; and EC, *E. coli*). Significantly higher SPAAC labeling using d-azido-alanine vs the parent d-alanine was seen for *S. aureus* (6.2 fold, *N* = 3, *P* = 0.00006), *L. monocytogenes* (1.6 fold, *N* = 3, *P* = 0.003), and *A. baumannii* (2.1 fold, *N* = 3, *P* = 0.0003).

We hypothesized that analogous to fluorescent SPAAC labeling, we could detect azide-modified bacteria using [^18^F]FB-sulfo-DBCO (Figure 4; Supporting Information Figure S5). As in the fluorescent case, the ligation of [^18^F]FB-sulfo-DBCO to azide-modified agarose beads was verified (Supporting Information Figure S4). We then tested the effect of d-azido-alanine concentration on [^18^F]FB-sulfo-DBCO SPAAC ligation in *S. aureus* cultures through a series of dilutions determining that 5 mM d-azido-alanine produced the highest signals (Supporting Information Figure S6). We then compared [^18^F]FB-sulfo-DBCO ligation in cultures treated with d-azido-alanine to those treated with 5 mM of l-azido-alanine (Figure 4B) showing significantly higher uptake for the d enantiomer (5.3-fold; *P* < 0.000001). The method was subsequently applied to longer side-chain lengths (Figure 4C), showing the highest SPAAC ligation for d-azido-alanine (*N* = 1), and to d-azido-alanine-OMe, which resulted in lower ligation versus the free carboxylate (5.6-fold; *P* = 0.000061) (Figure 4D). Finally, labeling experiments were conducted using the additional bacteria *Listeria monocytogenes*, *Pseudomonas aeruginosa*, *Acinetobacter baumannii*, and *Escherichia coli* with significantly more ligation observed in d-azido-alanine-treated cultures versus the d-alanine-treated controls for *L. monocytogenes* and *A. baumannii* (1.6-fold, *P* = 0.003 and 2.1-fold; *P* = 0.0003, respectively) (Figure 4E), with a similar pattern observed at lower d-amino acid concentrations (Supporting Information Figure S7).

### Dynamic PET/CT Imaging Analysis of [^18^F]FB-sulfo-DBCO in Normal Mice

**Figure 5:**
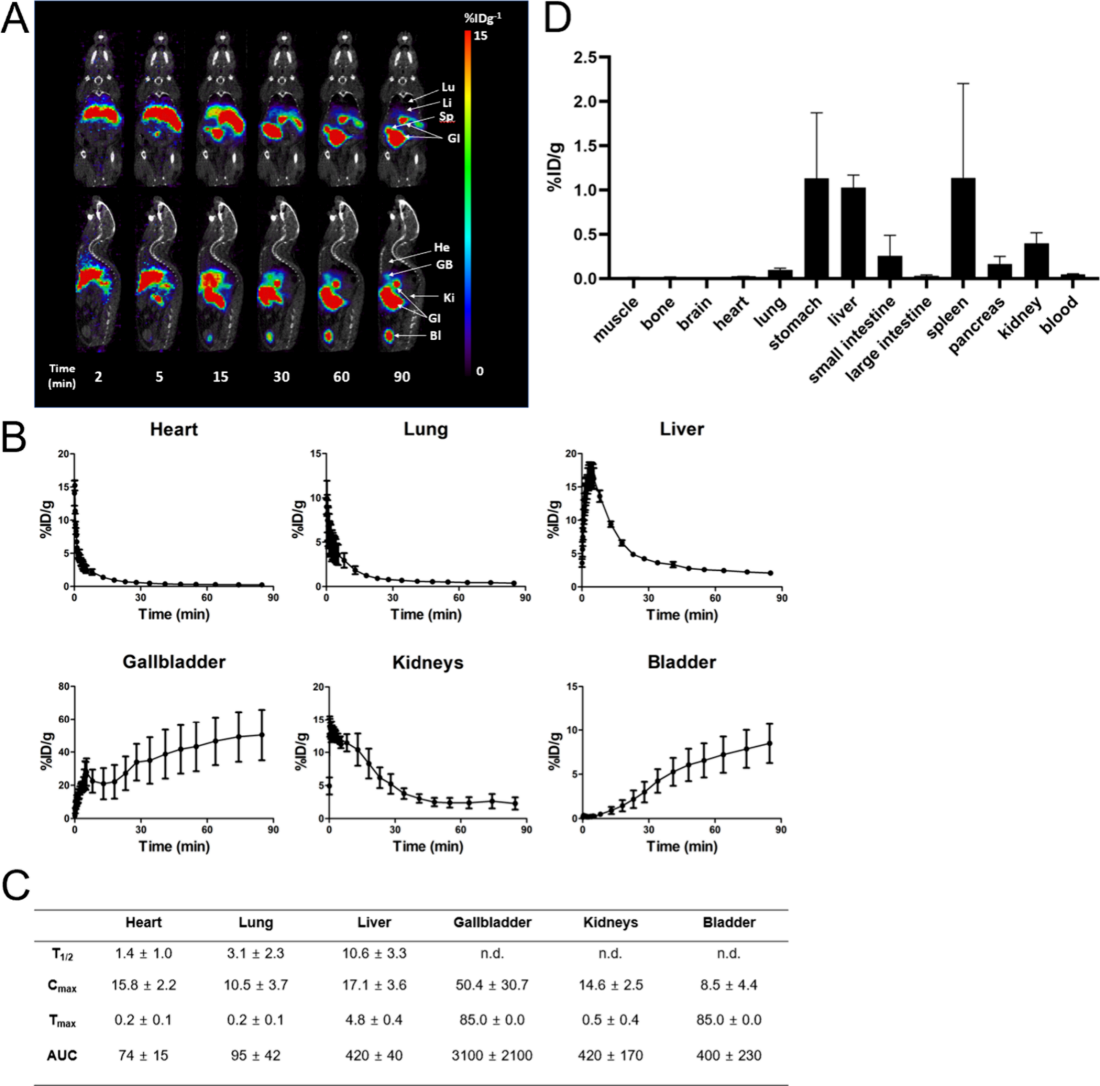
**Dynamic μPET/CT imaging analysis of [**^**18**^**F]FB-sulfo-DBCO in healthy mice.** (A) Representative time-course μPET/CT imaging of [^18^F]FB-sulfo-DBCO in a healthy mouse. He: heart, Lu: Lung, Li: liver; Sp: spleen, GI: Gastrointestinal, GB: gallbaldder, *Ki*: kidney, Bl: bladder. (B) Time-activity curves of [^18^F]FB-sulfo-DBCO in organs of healthy mice (*N* = 5). (C) Kinetic parameters of [^18^F]FB-sulfo-DBCO in healthy mice. *T*_1/2_: half-life (min), *C*_max_: peak concentration (%ID g^–1^), *T*_max_: time at *C*_max_ (min), AUC: area under the curve (%ID g^–1^ min), n.d.: not determined. (D) Biodistribution analysis of [^18^F]FB-sulfo-DBCO at 90 min obtained via tissue harvesting and gamma counting (*N* = 5).

The potential application of [^18^F]FB-sulfo-DBCO to *in vivo* imaging was analyzed in healthy mice. A cohort of healthy mice (*N* = 4) was studied using [^18^F]FB-sulfo-DBCO, with region of interest (ROI) analysis used to evaluate organ-specific tracer clearance (Figure 5) at early time points and *ex vivo* analysis (*N* = 5) at 90 min used to assess tracer retention in organs via harvesting and gamma counting. The initial peak uptake of [^18^F]FB-sulfo-DBCO was slightly higher in the heart (*C*_max_ = 15.8 ± 2.2% ID g^–1^, *T*_max_ = 0.2 ± 0.1 min) than in the lung (*C*_max_ = 10.5 ± 3.7% ID g^–1^, *T*_max_ = 0.2 ± 0.1 min). Clearance of [^18^F]FB-sulfo-DBCO from the lung (*T*_1/2_ = 3.1 ± 2.3 min) was relatively slower than that from the heart (*T*_1/2_ = 1.4 ± 1.0 min). After the initial uptake in the heart and lung, [^18^F]FB-sulfo-DBCO was cleared through the liver and kidneys via both hepatobiliary and urinary excretion. In the kidneys, the peak uptake of [^18^F]FB-sulfo-DBCO (*C*_max_ = 14.6 ± 2.5% ID g^–1^) was observed at the early time after intravenous injection (*T*_1/2_ = 0.5 ± 0.4 min), followed by excretion through the bladder over the time of imaging. Meanwhile, [^18^F]FB-sulfo-DBCO showed slightly higher uptake (*C*_max_ = 17.1 ± 3.6% ID g^–1^, *T*_max_ = 4.8 ± 0.4 min) in the liver than in the kidneys, with slow washout (*T*_1/2_ = 10.6 ± 3.3 min). [^18^F]FB-sulfo-DBCO accumulated in the liver was excreted through the gallbladder, spleen, and gastrointestinal system, as shown in the time-course whole-body distribution images (Figure 5A), suggesting that [^18^F]FB-sulfo-DBCO has a dominant biliary excretion pathway.

### Use of [^18^F]FB-sulfo-DBCO in *S. aureus*-Infected Mice Showed Low Signals in Affected Muscle

Motivated by several reports of imaging using pretargeted PET,^36,42^ we attempted *in vivo* detection of azide-modified bacteria using [^18^F]FB-sulfo-DBCO (Supporting Information Figure S8). An *S. aureus* culture was treated with d-azido-alanine as above and used to inoculate a murine cohort using previously reported methods.^19^ After 3 h, [^18^F]FB-sulfo-DBCO was injected intravenously as above, and the mice were studied after 30 min via tissue harvesting and *ex vivo* biodistribution analysis. The calculated retention of fluorine-18 by infected muscle was ∼0.1% ID/g (Supporting Information Figure S9), which was significantly lower than that we observed for d-amino acid-derived ^11^C tracers used successfully *in vivo* (∼7% ID/g for d-[3-^11^C]alanine).^19^

## Discussion

In this work, we explored a new technique for efficient screening of d-amino acid-derived fluorine-derived tracers using [^18^F]FB-sulfo-DBCO, a new water-soluble alkyne compatible with SPAAC recognition of azide-bearing peptidoglycan muropeptides. This fluorine-18 prosthetic group was readily synthesized from [^18^F]SFB, which is increasingly used in radiotracer synthesis. After validating our microbiology methods using fluorescent tools analogous to those previously reported, we showed that for several bacteria including *S. aureus*, [^18^F]FB-sulfo-DBCO labeling of 3-azido-d-alanine-treated cultures showed higher PET signals than those treated with 3-azido-l-alanine, 3-azido-l-alanine-OMe, or longer side-chain controls. These results, and those previously reported,^20^ have reinforced our thinking that for the incorporation of exogenous unnatural d-amino acids, resemblance to d-alanine itself is key. It is well-known that deviations from canonical amino acid structures (i.e., d-alanine, d-glutamate) impart changes to the biochemical behavior related to lipophilicity, size, and solvation. As expected, 3-azido-l-alanine-OMe showed poor [^18^F] signals likely reflecting low muropeptide incorporation, with the intriguing hypothesis that certain C-modified d-amino acids such as carboxamides actually increase bacterial uptake^24^ to be explored in future work. In addition, other d-amino acid scaffolds may be pursued. Previous work has shown that numerous exogenous d-amino acids can be incorporated into peptidoglycan, including d-methionine, d-phenylalanine, d-tryptophan, and d-valine.^43^ In addition, another carbon-11 PET infection imaging strategy used d-[3-^11^C]glutamine.^44,45^ Derivatives of these d-amino acids, therefore, represent additional routes for bioorthogonal bacterial detection using PET.

We also tested the new fluorine-18 SPAAC partner [^18^F]FB-sulfo-DBCO *in vivo* to predict how the reagent might be employed in future pathogen-specific imaging. Similar to previously reported aliphatic fluorine-18-labeled DBCO, [^18^F]FB-sulfo-DBCO showed a dominant hepatobiliary excretion pathway in normal mice. Meanwhile, we found that [^18^F]FB-sulfo-DBCO had lower background signals than the aliphatic fluorine-18-labeled DBCO likely due to the enhanced hydrophilicity of [^18^F]FB-sulfo-DBCO by the insertion of a sulfonic acid group. In addition, aromatic fluorine-18-labeled [^18^F]FB-sulfo-DBCO showed significantly lower bone uptake than that of the aliphatic fluorine-18-labeled DBCO.^46^ We found that [^18^F]FB-sulfo-DBCO had weak background signals in normal mice. The accumulation of [^18^F]FB-sulfo-DBCO was less than 1% ID/g in most organs, with the highest uptake seen in the stomach, liver, and spleen, which would potentially limit the potential detection of bacteria in those organs. We also observed weak signals in tissues infected with azide-modified *S. aureus*. For successful pretargeting, we will explore faster bioorthogonal cycloadditions for more sensitive bacterial detection.^32^ For example, larger rate constants are observed using inverse electron demand Diels–Alder (iEDDA) click chemistry (1–10^6^ M^–1^ s^–1^),^47^ which may enhance the *in vivo* feasibility of pretargeted PET imaging. In the future, we anticipate that *in vivo* bacterial detection will be possible using tetrazine-modified bacteria-specific substrates imaged using fluorine-18-labeled dienophiles.

To our knowledge, these are the first data supporting a pretargeted infection imaging approach using PET-compatible radiopharmaceuticals. Several analogous chemical biology approaches have been previously reported in the fluorescence literature, using cell wall-specific tools. In addition to the muropeptide-targeted azide–alkyne approach previously discussed, the alternating *N*-acetyl muramic acid/*N*-acetyl glucosamine (NAM/NAC) sugar backbone of peptidoglycan has also been explored by the Grimes group for detecting the bacterial cell membrane, via both azide and alkyne modification of the N-acetyl moiety of NAM.^28^ Several subsequent manuscripts reported additional NAM derivatives, potentially supporting modification of this scaffold for PET.^29^ Gram-negative organisms have been labeled via azide-modified 3-deoxy-d-manno-octulosonic acid (KDO), a component of lipopolysaccharide (LPS) in the bacterial outer membrane.^31^ Sensing LPS or “endotoxin” might have a significant impact on human health, based on both Gram-negative specific recognition and the interactions between LPS and the immune system resulting in fever and occasionally septic shock.^48^ The discovery of these chemical biology tools has also significantly accelerated our understanding of microbe behavior.

A major obstacle to PET tracer discovery is the low throughput of radiosynthesis and radiopharmaceutical validation, with exploration of a new tracer concept typically conducted with several steps in series (precursor synthesis, variable radionuclide incorporation, lengthy HPLC characterization and purification, stability testing, and biologic validation). Therefore, PET methods compatible with high-throughput analysis are critical for the efficient discovery of new pharmaceuticals. The fluorescence literature provides numerous clues as to bacteria-tolerated unnatural d-amino acids, but we wanted to develop a method that would more efficiently generate [^18^F] radiopharmaceuticals compatible with PET imaging. In particular, modifications of the d-amino acid side chain and C-terminus might result in tracers with enhanced microbial sensitivity that are potentially compatible with a pretargeting imaging method. We anticipate that [^18^F]FB-sulfo-DBCO will have further use in discovering bacteria-specific tools and new radiopharmaceuticals for other applications.

## Conclusions

In conclusion, we have synthesized and tested the new water-soluble [^18^F]SFB-derived strained cyclooctyne radiotracer [^18^F]FB-sulfo-DBCO for SPAAC labeling of the bacterial cell wall. Bacteria pretreated with azide-bearing d-amino acids including 3-azido-d-alanine showed increased [^18^F]FB-sulfo-DBCO labeling versus cultures treated with the cognate l-amino acid or a C-terminal modified derivative. *In vivo*, [^18^F]FB-sulfo-DBCO showed rapid clearance and low background with no evidence of defluorination. Future efforts will focus on optimizing this pretargeting imaging approach and developing [^18^F]-labeled tracers with minimal structural/steric alterations versus the d-alanine parent.

## Materials and Methods

### Radiosynthesis of [^18^F]FB-sulfo-DBCO

[^18^F]SFB was synthesized using a procedure reported by Nagachinta et al.^39^ To a 4 mL borosilicate vial containing [^18^F]SFB (370–555 MBq) were added a PTFE stir bar, Et_3_N (25 μL), DMF (500 μL), and Sulfo DBCO amine precursor (5 mg). The mixture was stirred at 40 °C for 20 min, then diluted with H_2_O before purification via semiprep HPLC using a Phenomenex Luna C18 column, 10 mm (40% MeCN/60% H_2_O + 0.1% TFA). [^18^F]FB-sulfo-DBCO was isolated in a 2–3 mL fraction. The fraction was diluted with H_2_O (30 mL) before being passed through a Sep-pak light C18 Cartridge at 5 mL/min. An additional 10 mL of H_2_O was used to wash the cartridge. After the cartridge was flushed with air, the product was eluted using EtOH (0.5 mL) for direct formulation prior to *in vitro* or *in vivo* use. Isolated [^18^F]FB-sulfo-DBCO had RCY = 50.8% ± 9.3 n.d.c (*n* = 12), RCP ≥ 99%, and *A*_m_ = 2.9 ± 0.6 GBq/μmol. The chemical purity of [^18^F]FB-sulfo-DBCO was verified by analytical HPLC. Isolation and characterization of the cold [^19^F]FB-sulfo-DBCO standard are described in the Supporting Information.

### Agarose Labeling

To test the feasibility of SPAAC labeling with fluorophore and PET alkyne in our optimized assay, agarose controls were treated with DBCO partners. Commercially available azide and alkyne agaroses (Click Chemistry Tools, Scottsdale, AZ) were aliquoted to microcentrifuge tubes and diluted 1:10 in PBS. The agaroses were incubated with each respective DBCO partner and rapidly agitated for 1 h at 37 °C. Agaroses were thoroughly resuspended, and 100 μL aliquots were centrifuged at 12,000*g* for 6 min in spin filters lined with 0.22 μm cellulose acetate filters. The filter cartridges were washed with wash buffer (2% v/v FBS, 0.05% v/v Tween-20 in DPBS), and SPAAC labeling for each agarose and corresponding filtrate were measured separately using a fluorescent plate reader (Flexstation III, Molecular Devices LLC, US) or an automated gamma counter (Hidex, Turku, Finland).

### *In Vitro* Experiments

All bacterial studies were supported by an approved UCSF biological use authorization (BUA) protocol. All bacterial strains were purchased from American Type Culture Collection (ATCC) and were aerobically grown using the conditions outlined in the Supporting Information.

### Azide Incorporation

Bacteria cultures (10 mL) were subcultured from an overnight culture and grown to exponential growth phases. A 4.5 mL portion was subsequently incubated with a final concentration of 5 mM d-azido-alanine at 37 °C for 1 h rapidly agitated at 180 rpm. These cultures (5 mL) were pelleted by centrifugation at 12,000*g* for 5 min and washed with wash buffer (defined above) 5 times. Azide-incubated bacterial pellets were resuspended in 500 μL of PBS for further SPAAC partner labeling.

### Optical SPAAC Labeling

Azide-labeled bacteria (500 μL in PBS) were aliquoted and incubated with cyclooctyne fluorophore (1% in PBS) derivates: DBCO-MB-543, Cy 5.5 cyclooctynefluorophore, AlexaFluor Dye 488 DBCO (Click Chemistry Tools, Scottsdale, AZ). The cultures were rapidly agitated at 180 rm at 37 °C for 1 h and pelleted at 8000*g* for 5 min. The pellet was reconstituted, washed with the same wash buffer 3 times, and reconstituted in PBS. Cultures were added to a 96-well plate and analyzed on a fluorescent plate reader (Flexstation III, Molecular Devices LLC, US).

### SPAAC Radiolabeling

Radiotracer SPAAC ligation assays were performed by adding [^18^F]FB-sulfo-DBCO (0.89 MBq/experiment) to previously azide-incubated bacterial cultures (500 μL). The ligation was rapidly agitated at 180 rpm at 37 °C for 1 h. As a biological control, bacterial cultures were grown under similar conditions and treated with d-alanine following the aforementioned protocol. Bacterial cultures (100 μL aliquots) were centrifuged at 12,000*g* for 6 min in spin filters lined with a 0.22 μm cellulose acetate filter. The filter cartridges were washed with the same wash buffer, and the activity for each bacterial pellet and filtrate was measured separately using an automated gamma counter (Hidex) and normalized to the optical density (GENESYS 20 Visible Spectrophotometer).

### *In Vitro* Studies

#### μPET/CT Imaging Studies of [^18^F]FB-sulfo-DBCO

All μPET/CT imaging studies were conducted with the Siemens Inveon micro PET-CT scanner (Siemens, Erlangen, Germany). All animal studies were approved by the Institutional Animal Care and Use Committee at UCSF and performed in accordance with the UCSF guidelines. Healthy CBA/J mice (female, 9–11 weeks old, 20–24 g) were used for all of the experiments. Animals were anesthetized using 5% isofluorane during radiotracer tail vein catheter injections and μPET/CT imaging.

All studies used a 90 min whole-body dynamic PET acquisition of animals (*N* = 4) obtained with 34 frames (10s × 11, 20s × 11, 300s × 5, 420s × 4, 630s × 3 frames, respectively) with [^18^F]-Sulfo-DBCO (200 ± 21 MBq, 100 μL) via tail vein injection, followed by a micro-CT scan for 10 min. All data were reconstructed into three-dimensional dynamic PET images and were coregistered with CT images using open-source AMIDE software.

#### Image Analysis

AMIDE open-source software^33^ was used to analyze all imaging data. Volumes of interest (VOIs) were manually drawn for each organ (heart, lung, liver, gallbladder, kidney, bladder) to obtain the radiotracer’s biodistribution profile in healthy mice. Radioactivity in VOIs at each time point was expressed as the percent injected dose per g (%ID g^–1^) and was used to generate time-activity curves (TACs). Kinetic parameters of radiotracers in each organ were calculated from the TACs by fitting a single exponential curve using GraphPad Prism v9.0 software (GraphPad Software Inc., San Diego, California, USA) as follows: half-life (*T*_1/2_), peak concentration (*C*_max_), time at *C*_max_ (*T*_max_), and area under the curve (AUC).

### Data Analysis and Statistical Methods

Data was represented in graphs depicted with error bars corresponding to the standard error of the mean. *In vitro S. aureus* data were obtained with 3 biological replicates per experiment, and *in vivo* data were obtained with 5 biological replicates per experiment, with the presumed sources of error instrumental/procedural and biological diversity. All statistical analyses of *in vitro* data were performed using Microsoft Excel, program language R (https://www.R-project.org/), and Prism software version v9.0 (GraphPad, CA). The *in vitro* data reported represent 3 replicates per experiment. Data was analyzed using one-way analysis of variance tests (ANOVA) and/or unpaired two-tailed Student’s *t* test. Micro PET/CT data was analyzed using open-source software AMIDE, and %ID g^–1^ was used for quantitative comparison. All *in vivo* organ harvesting data represent 5 replicates per experiment. A 95% confidence interval was used to distinguish significant differences between data points.

## Supporting Information Available

Detailed information regarding microbiology, radiosynthesis, and several *in vitro* studies not reported in the main text (PDF)
