# Supplementary material for: Bioorthogonal Radiolabeling of Azide-Modified Bacteria Using [18F]FB-sulfo-DBCO
Source: Bioconjug Chem. 2024 Mar 14;35(4):517–27. doi: 10.1021/acs.bioconjchem.4c00024 (PMC11036355; doi:10.1021/acs.bioconjchem.4c00024)
Supplement: Supplementary file 1 — bc4c00024_si_001.pdf [file bc4c00024_si_001.pdf]

Supporting Information for:

Bioorthogonal radiolabeling of azide-modified bacteria using [^{18}F]FB-sulfo-DBCO

Aryn A. Alanizi[1], Alexandre M. Sorlin[1], Matthew F. L. Parker[1,2] Marina López-Álvarez [1], Hecong Qin[1], Sang Hee Lee[1], Joseph Blecha[1], Oren S. Rosenberg[3], Joanne Engel[3], Michael A. Ohliger[1,4], Robert R. Flavell[1], David M. Wilson[1*]

[1]Department of Radiology and Biomedical Imaging
University of California, San Francisco
San Francisco, CA 94158, USA

[2]Department of Psychiatry
Renaissance School of Medicine at Stony Brook University
Stony Brook, NY, 11794 USA

[3]Department of Medicine
University of California, San Francisco
San Francisco, CA 94158, USA

[4]Department of Radiology
Zuckerberg San Francisco General Hospital
San Francisco CA 94110, USA

Table of Contents

A. **Supplemental Figures**……………………………………………………………………2
B. **Synthetic Procedures**…………………………………………………………………...…...9
C. **Radiochemistry**……………………………………………………………………..…………12
D. **References**……………………………………………………………………………………..14

A. Supplemental Figures

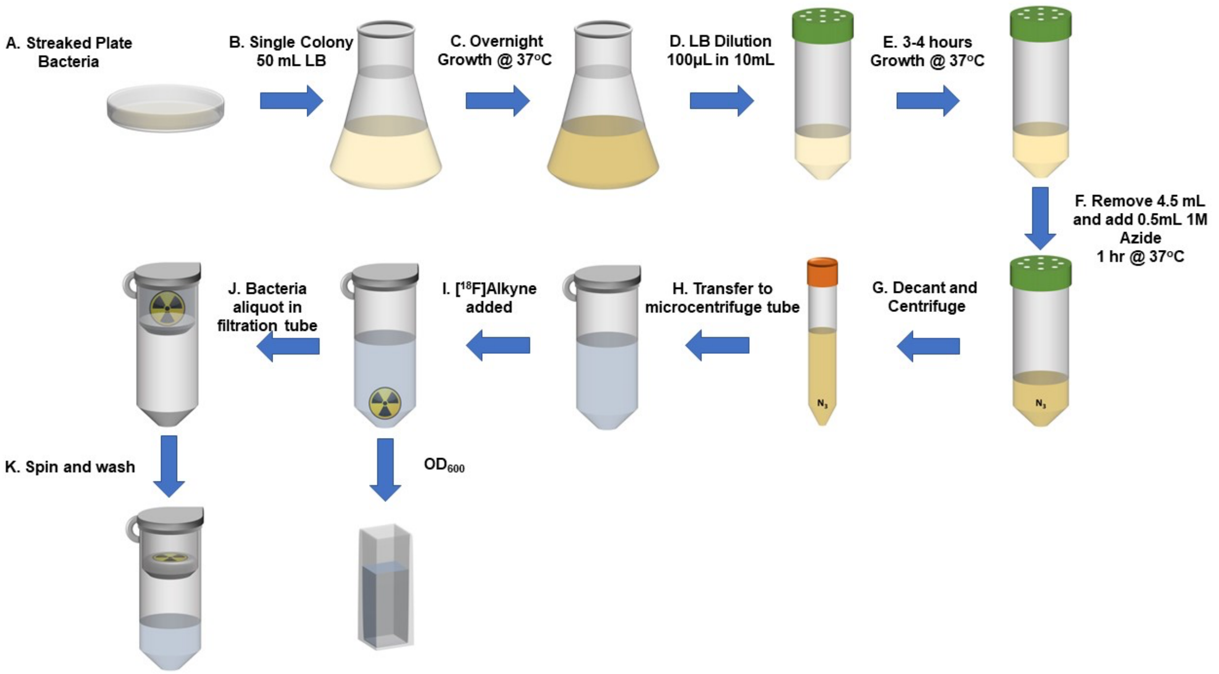

Figure S1. Optimized high-throughput assay for fluorescence/radiolabeled SPAAC. Workflow for high-throughput *in vitro* assay for labeling bacterial cell wall with azide-derived alanine derivatives followed by ligation with positron emitting or fluorophore cyclooctyne. OD_{600} measurements were serially acquired to monitor bacterial growth state.

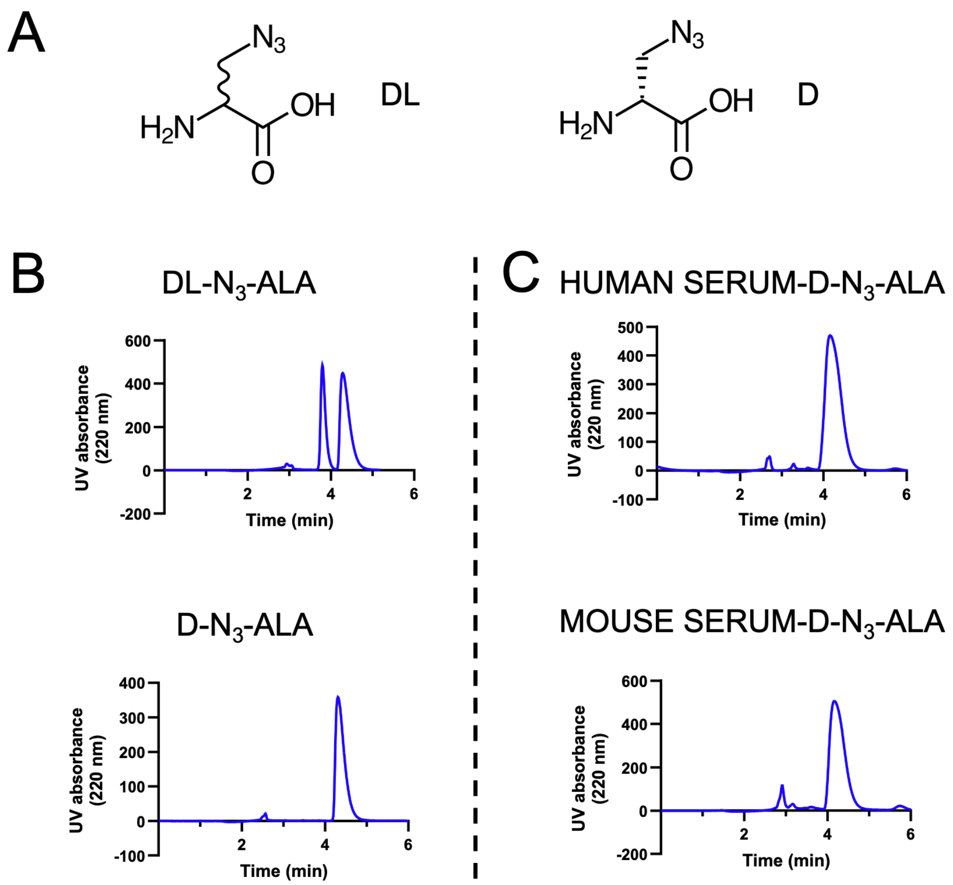

Figure S2. Stability data for 3-azido-D-alanine. We first demonstrated that racemic azido-alanine could be resolved using a chiral stationary phase HPLC (left). In all cases the azido-alanine sample was injected on an Astec Chirobiotic TAG column with a mobile phase of 10% MeOH/90% H2O @ 1 mL/min, and a 220 nm observation wavelength. On the left, 3-azido-D-alanine could be easily resolved from its L-counterpart. On the right, 3-azido-D-alanine was incubated @ 37 °C in either human or mouse serum for 3 hours showing absence of racemization under physiologic conditions.

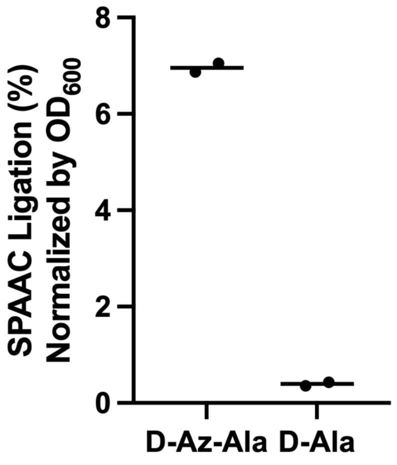

Figure S3. SPAAC fluorophore labeling of *S. aureus* with AF 488. Quantification of signals generated by AF 488 ligation to *S. aureus* treated with 3-azido-D-alanine and D-alanine were measured to test our high-throughput SPAAC assay. Bacteria were separated from media to measure SPAAC fluorescence. Analysis of pellet-retained fluorescent signal versus filtrate (N = 2, data points are represented in relation to their mean).

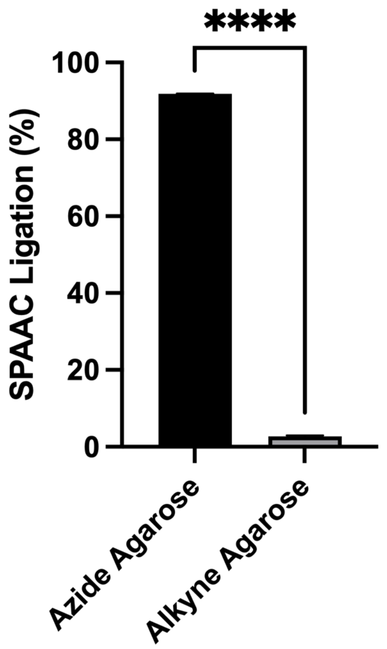

Figure S4. Analysis of azide and alkyne studded agarose beads [^{18}F]FB-sulfo-DBCO radioactive signal versus filtrate (N = 3, $p < 0.0001$).

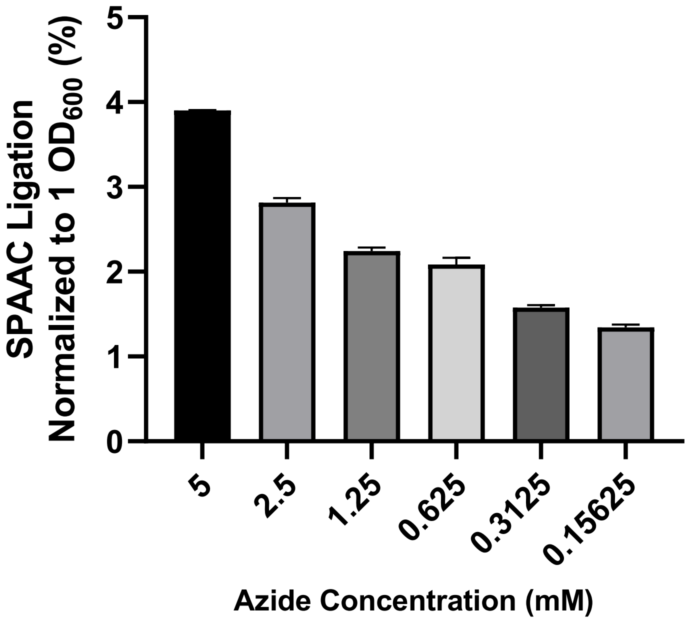

Figure S5. The effect of 3-azido-D-alanine concentration on SPAAC ligation using [^{18}F]FB-sulfo-DBCO (N = 3 for all experiments). Increasing ^{18}F incorporation was observed when higher concentrations of 3-azido-D-alanine were incubated with *S. aureus*.

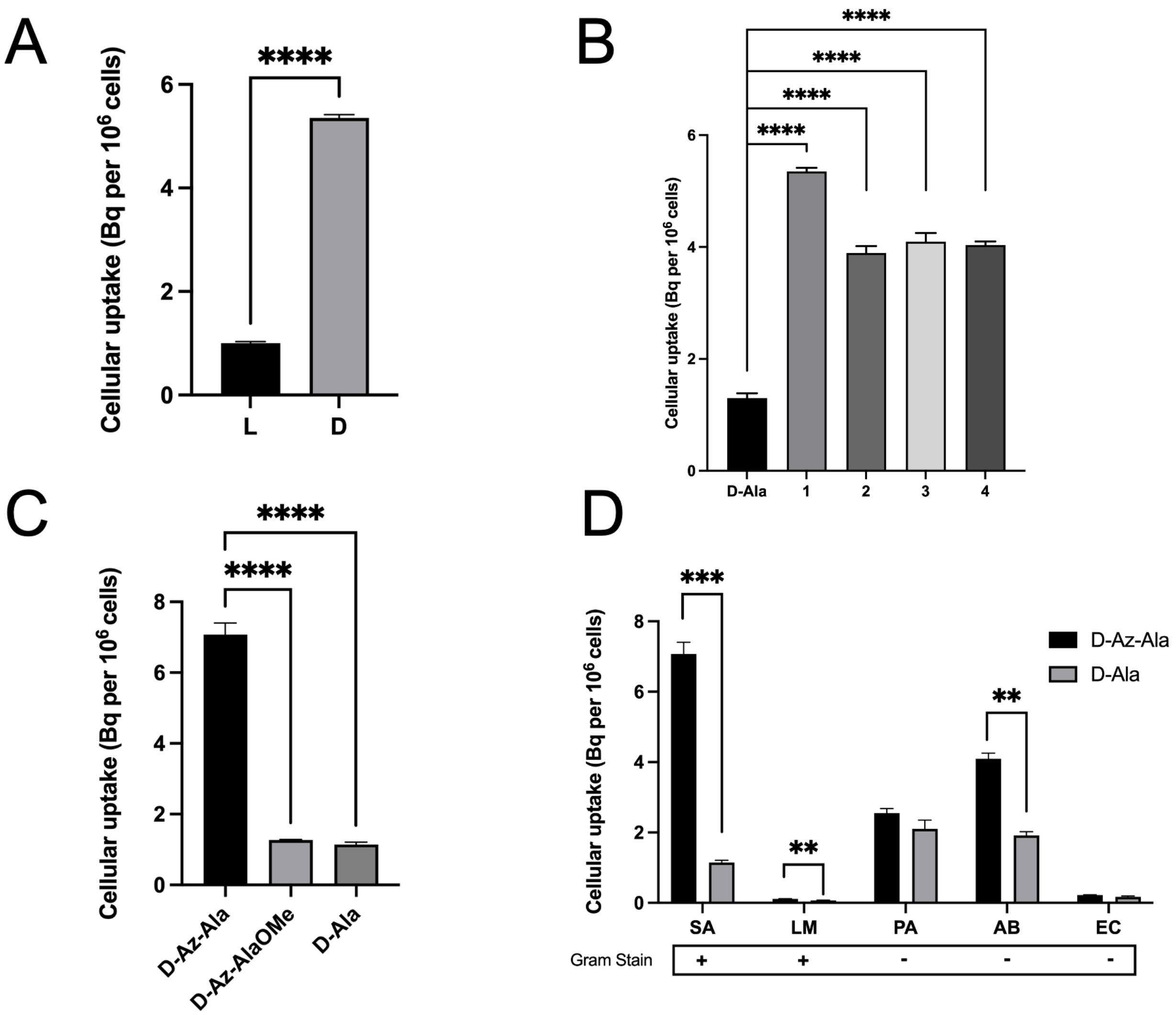

Figure S6. *In vitro* data using[^{18}F]FB-sulfo-DBCO in azide-modified bacteria presented in retained radioactivity per colony performing units (CFU's). N = 3 for all experiments. (A) Effect of pre-incubation with L- versus 3-azido-D-alanine on [^{18}F]FB-sulfo-DBCO ligation. (B) Effect of side-chain length showing the highest signals for N=1 (3-azido-D-alanine). (C) Effect of C-terminal modification via methyl ester. (D) Comparison of different pathogens pre-incubate with 3-azido-D-alanine.

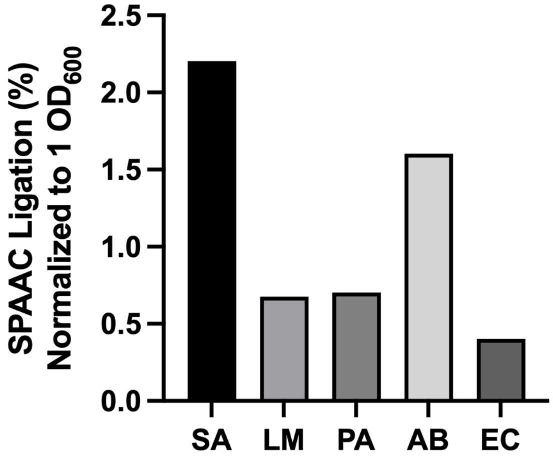

Figure S7. Analogous to the results using 5 mM 3-azido-D-alanine reported in the main manuscript, we performed a 2 mM study with an identical pattern observed among pathogens (N = 1). Lower SPAAC ligation was observed for all all pathogens. (SA, *S. aureus*; LM, *L. monocytogenes*; PA. *P. aeruginosa*; AB, *A. baumannii*; EC, *E. coli*).

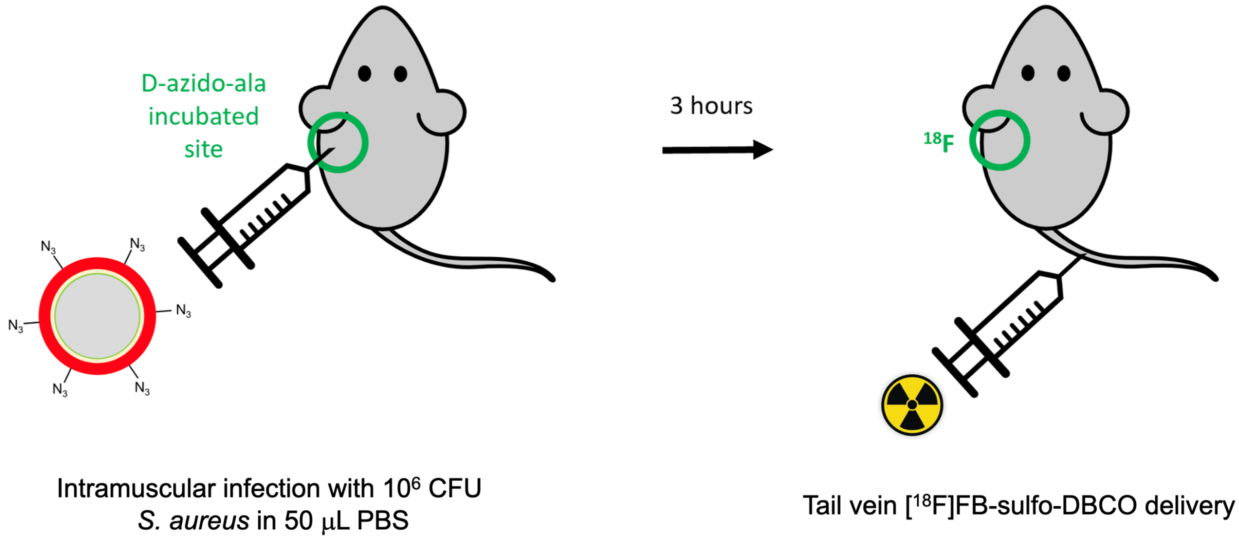

Figure S8. Pilot myositis "pre-incubation" pre-targeting *in vivo* schema. A pilot myositis infection model (N = 5) was established with intramuscular inoculation of *S. aureus* (10^6 CFU in PBS vehicle) incubated with 3-azido-D-alanine following the established high-throughput *in vitro* assay. [^{18}F]FB-sulfo-DBCO (200 ± 21 MBq, 100 μL in PBS) was intravenously delivered via tail vein injection three hours post inoculation.

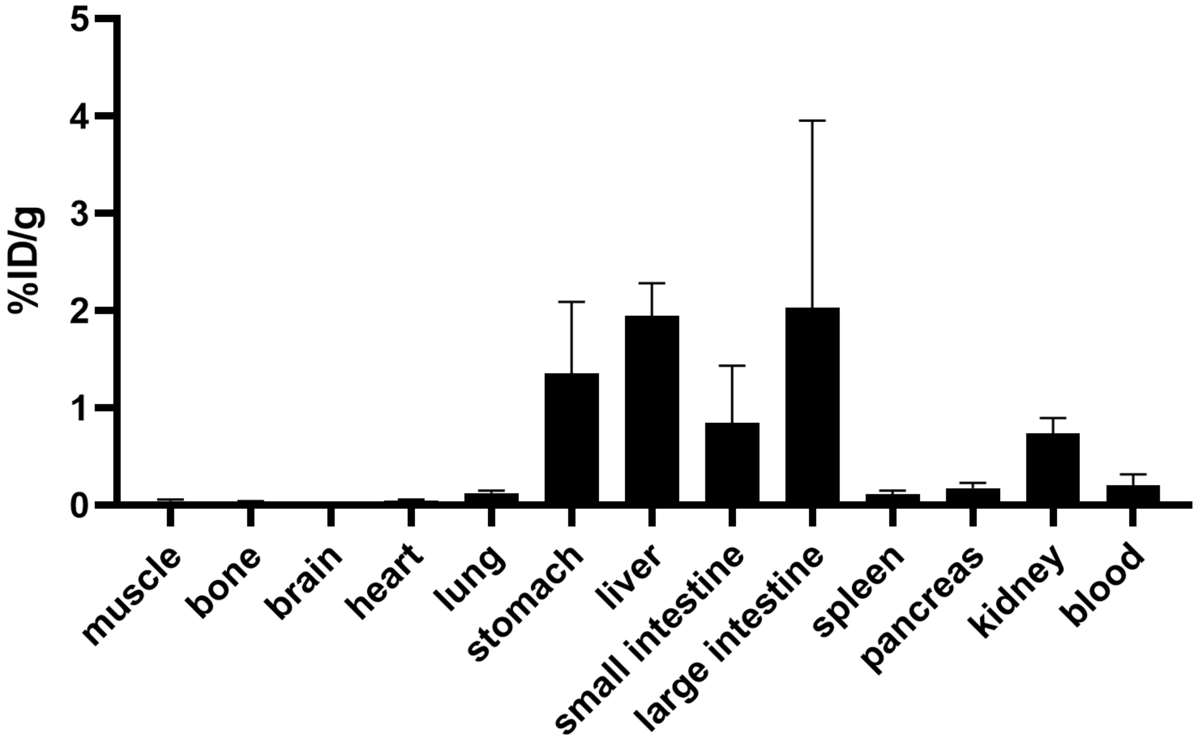

Figure S9. Pre-incubation biodistribution analysis of [^{18}F]FB-sulfo-DBCO at 90 minutes obtained via tissue harvesting and gamma counting (N = 5). Infected muscular incorporation of [^{18}F]FB-sulfo-DBCO reflects similar levels in healthy mice. Signals in infected muscle were ~ 0.1 %ID/g.

Table S1. Bacterial strains

The bacterial strains and growth conditions included in this study are listed in the table below.

**Strain**	**Phenotype or Genotype**	**Growth Temperature (°C)**	**Media**	**Source/ Reference**
S. aureus	Wild-type	37	Lysogeny Broth	ATCC 12600
L. monocytogenes	Wild-type	37	Brain Heart Infusion Broth	ATCC 15313
E. coli	Wild-type	37	Lysogeny Broth	ATCC 25922
P. aeruginosa PA01	Wild-type	37	Lysogeny Broth	ATCC 10154
A. baumannii	Wild-type	37	Lysogeny Broth	ATCC 19606

B. Synthetic Procedures

B.1. General:

All chemical reagents were purchased from commercial sources (Acros Organics, Alfa Aesar, AK Scientific, Click Chemistry Tools, & Sigma-Aldrich) and used without further purification unless otherwise stated. All separatory cartridges were purchased from Waters. Reactions were monitored by thin layer chromatography (TLC) on precoated (250 μm) silica gel 60 F254 aluminum sheets and visualized under a UV-254 lamp followed by staining with potassium permanganate. Flash chromatography was performed on silica gel (60A pore size). ^{1}H, ^{13}C and ^{19}F NMR spectra were obtained on a Bruker Avance III HD 400 mHz instrument at the UCSF Nuclear Magnetic Resonance Laboratory and data were processed using MestReNova. Abbreviations are as follows: s (singlet), d (doublet), t (triplet), q (quartet), m (multiplet). High resolution mass spectra (HRMS) services were provided by University of California, Berkeley Spectrometry Facility. The ^{18}F labeled compounds were characterized by analytical HPLC, using a Waters pump equipped with a manual Rheodyne injector (1 mL loop), a UV detector and a RAD detector. Reversed-phase chromatography used a Phenomenex Luna C18 column stationary phase, at a flowrate of 1 mL/min, and a 254 nm observation wavelength. The radioactivity of the bacterial pallets and filtrate were counted on a γ counter (Hidex Automatic Gamma Counter).

B.2. Synthesis of [^{19}F]FB-sulfo-DBCO standard

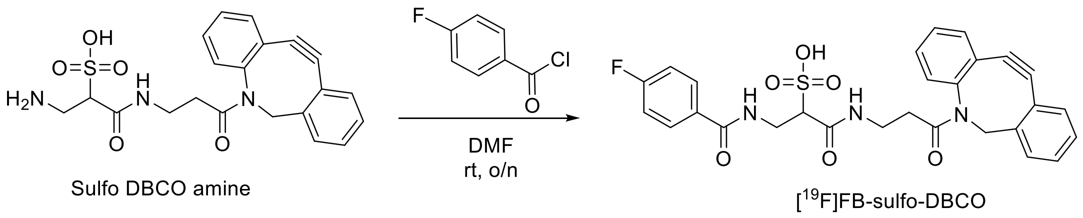

In a 4 mL borosilicate vial containing PTFE stir bar, a 0.1 M solution of 4-fluorobenzoyl chloride (98%, Sigma Aldrich) (9.27 mg, 0.0585 mmol) and Sulfo DBCO amine (Click Chemistry Tools) (25 mg, 0.0585 mmol) were added and mixed with 0.5 mL of DMF and stirred at room temperature overnight. The mixture was diluted with MeCN and purified via semi prep HPLC using Phenomenex Luna C18 column, 10 mm (40% MeCN/60 % H_2O +0.1% TFA) to yield compound [^{19}F]FB-sulfo-DBCO.

^{1}H NMR (400 MHz, MeOD) δ 7.86 (ddd, *J* = 25.4, 8.7, 5.3 Hz, 1H), 7.67 – 7.13 (m, 5H), 5.13 (d, *J* = 14.0 Hz, 1H), 3.92 (dd, *J* = 13.4, 5.7 Hz, 1H), 3.85 – 3.76 (m, 1H), 3.69 (d, *J* = 13.9 Hz, 1H), 3.66 – 3.56 (m, 1H), 3.21 – 3.12 (m, 1H), 2.60 (dt, *J* = 23.3, 7.6 Hz, 1H), 2.11 – 1.92 (m, 1H).

^{13}C NMR (100 MHz, MeOD) δ 173.3, 169.0, 167.5, 165.0, 152.6, 149.4, 133.5, 131.9, 130.9, 130.7, 130.1, 129.6, 129.2, 128.9, 128.0, 126.7, 126.4, 116.5, 116.3, 115.6, 108.8, 66.1, 65.9, 56.7, 40.2, 40.1, 36.9, 36.7, 35.2.

^{19}F NMR (376 MHz, MeOD) δ -110.73 (s).

HRMS (ESI) m/z calculated for $C_{20}H_{20}BrF_2NO_2$ (M+H) 549.14, found 549.14.

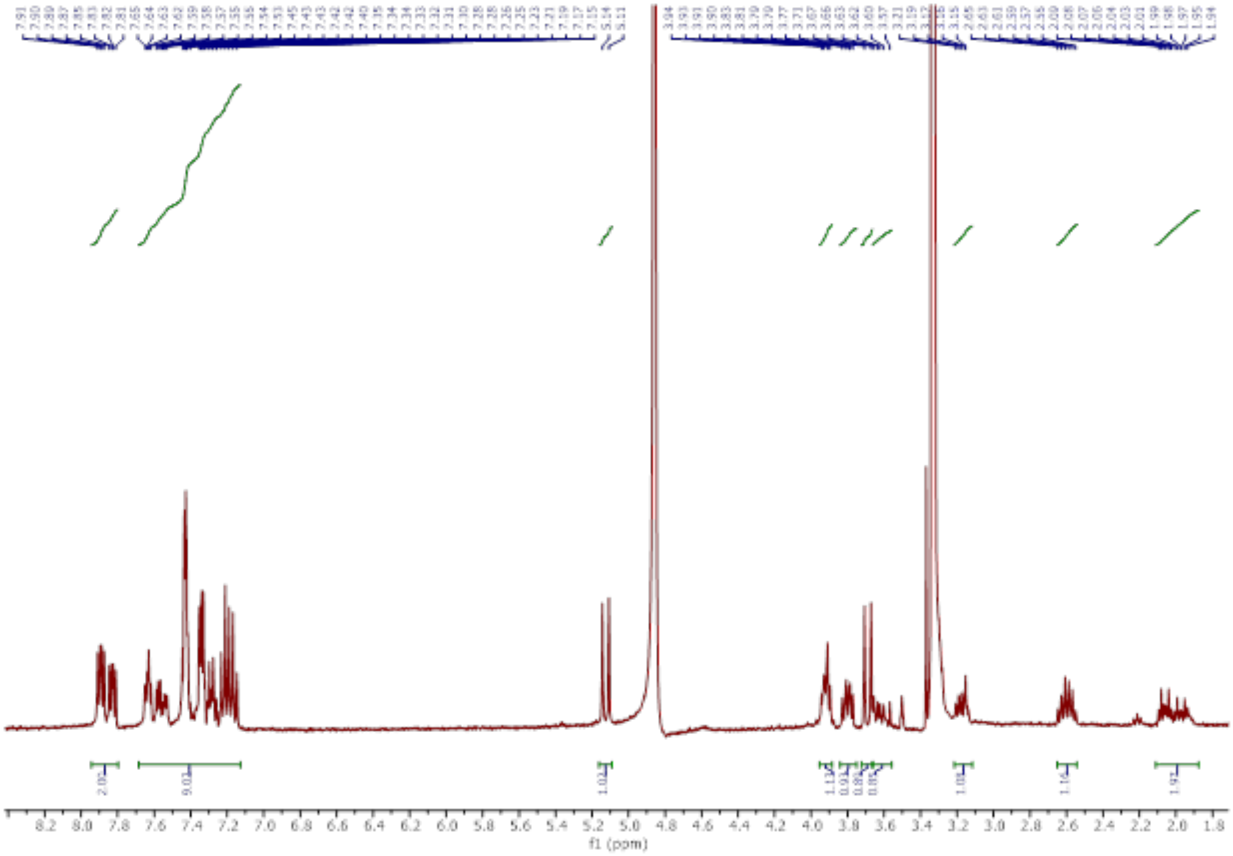

Figure B.2.1. ^{1}H NMR of [^{19}F]FB-sulfo-DBCO in MeOD.

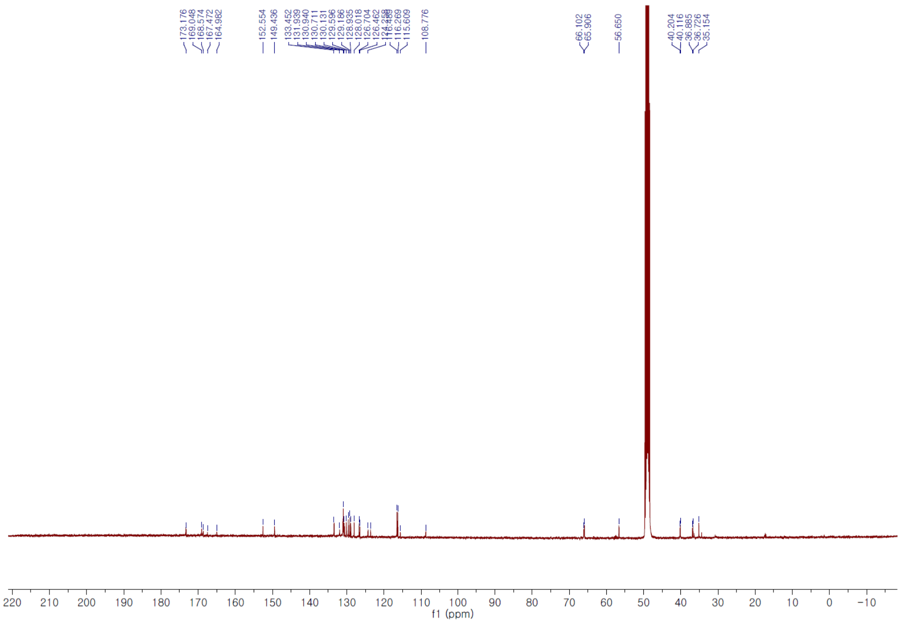

Figure B.2.2. ^{13}C NMR of [^{19}F]FB-sulfo-DBCO in MeOD.

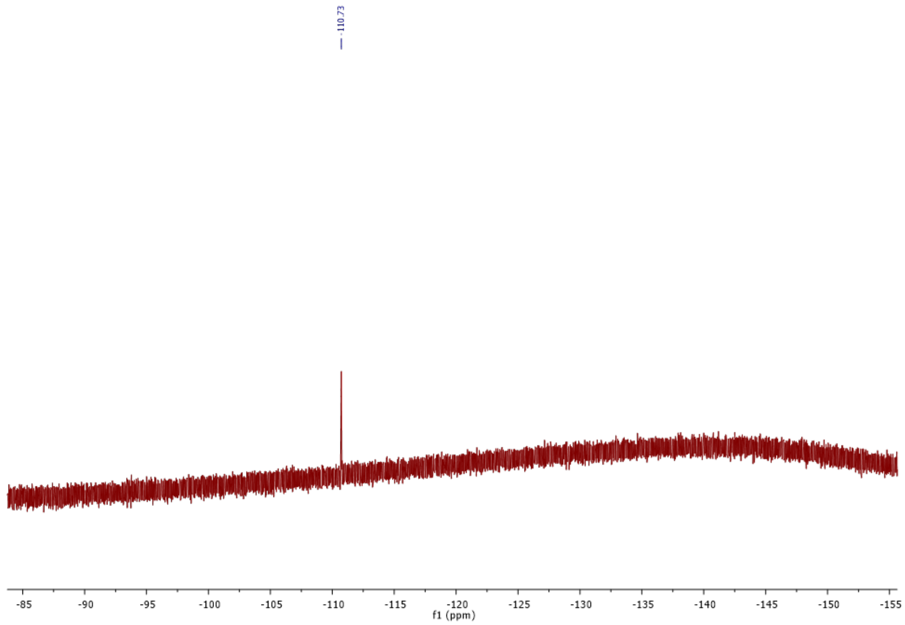

Figure B.2.3. ^{19}F NMR of [^{19}F]FB-sulfo-DBCO in MeOD.

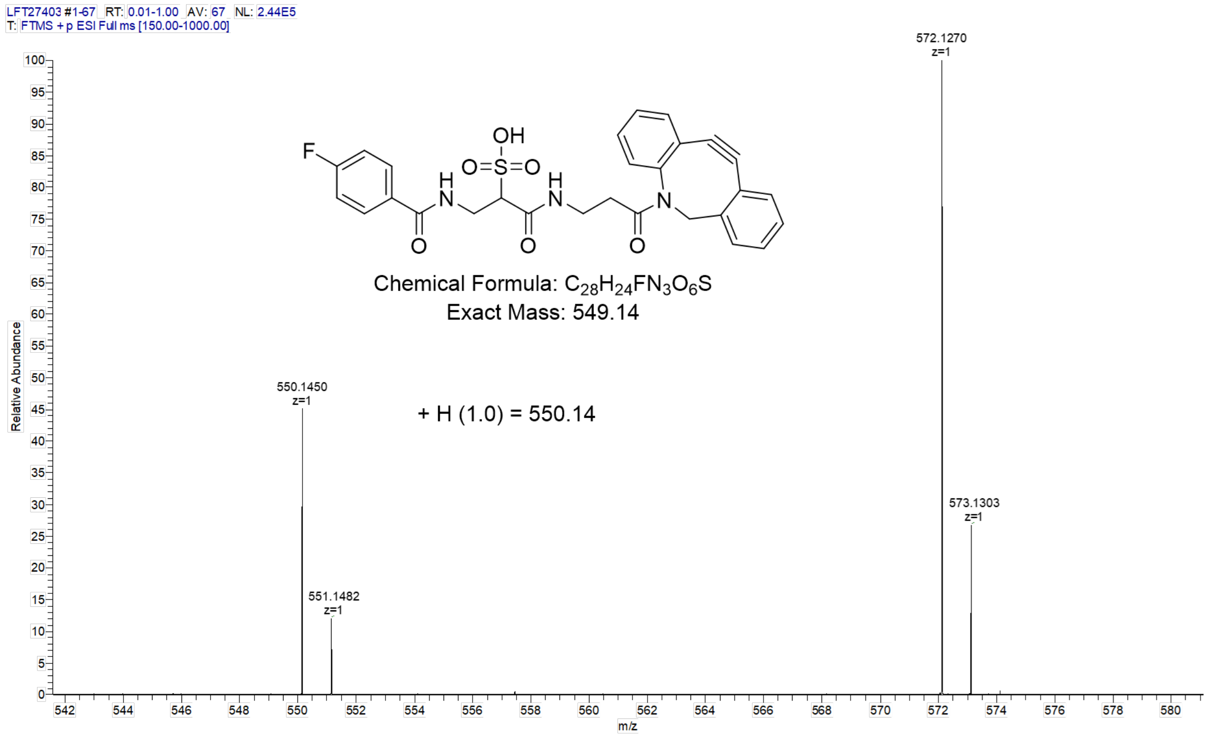

Figure B.2.4. HRMS of [19F]FB-sulfo-DBCO. HRMS (ESI) m/z calculated for $C_{28}H_{24}FN_3O_6S$ (M+H) 550.14, found 550.14.

C. Radiochemistry

C.1. Detailed radiosynthesis of [18F]FB-sulfo-DBCO

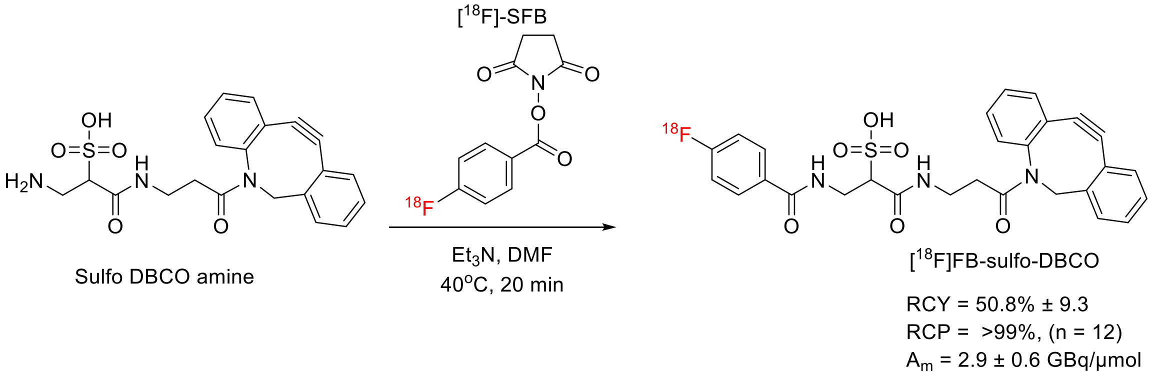

In a 4 mL borosilicate vial containing [18F]SFB (10-15 mCi) was added PTFE stir bar, Et_3N (25 μL), DMF (500 μL) and precursor Sulfo DBCO amine (5 mg). The mixture was stirred at 40°C for 20 min, then diluted with H_2O before purification via semi prep HPLC using Phenomenex Luna C18 column, 10 mm (40% MeCN/60 % H_2O +0.1% TFA). [18F]FB-sulfo-DBCO was isolated in 2-3mL fraction. The fraction was diluted with H_2O (30 mL) before being passed through Sep-

pak light C18 Cartridge at 5 mL/min. An additional 10 mL of H_2O was used to wash the cartridge. After flushing the cartridge with air, the product was eluted using EtOH solution (0.5 mL) for direct formulation before use *in vitro* or *in vivo*. [^{18}F]FB-sulfo-DBCO (RCY = 50.8% ± 9.3 decay corrected (n = 12), RCP = >99%, Am = 2.9 ± 0.6 GBq/µmol). Chemical purity of [^{18}F]FB-sulfo-DBCO was verified by analytical HPLC.

HPLC analysis of [^{18}F]FB-sulfo-DBCO:

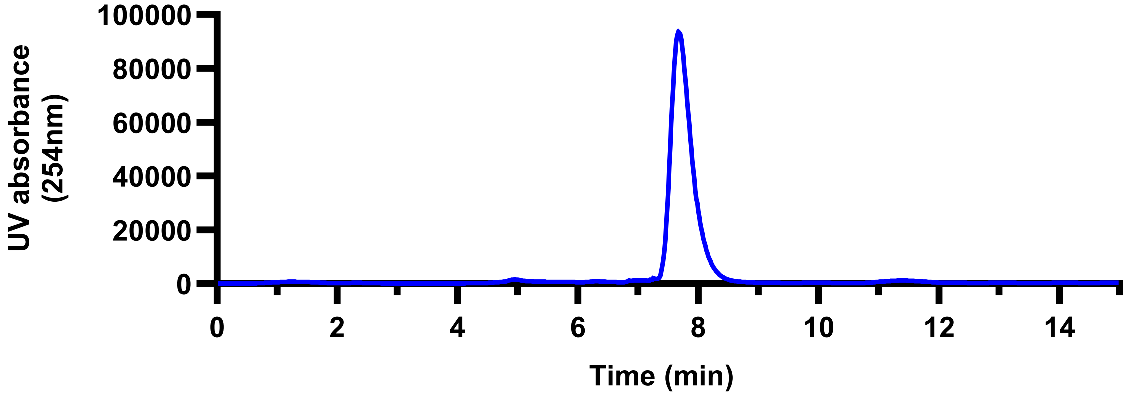

Figure C.2.1. HPLC analysis (UV detection) of [^{19}F] standard FB-sulfo-DBCO

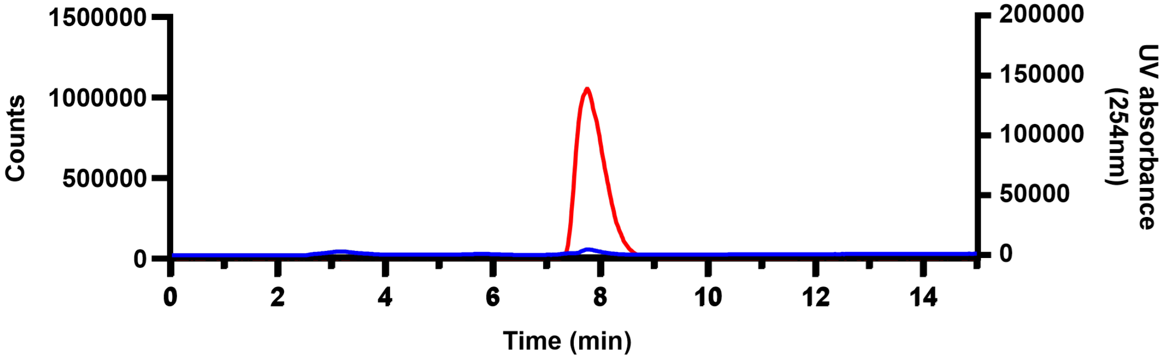

Figure C.2.1. HPLC analysis (RAD and UV detection) of isolated [^{18}F]FB-sulfo-DBCO

References:

(1) Yang, W., Mou, T., Peng, C., Wu, Z., Zhang, X., Li, F., and Ma, Y. (2009) Fluorine-18 labeled galactosyl-neoglycoalbumin for imaging the hepatic asialoglycoprotein receptor. *Bioorg. Med. Chem. 17*, 7510–7516.

(2) Qin, L., Hu, B., Neumann, K. D., Linstad, E. J., McCauley, K., Veness, J., Kempinger, J. J., and DiMagno, S. G. (2015) A Mild and General One-Pot Synthesis of Densely Functionalized Diaryliodonium Salts. *European J. Org. Chem. 2015*, 5919–5924.

(3) Wang, L., Zha, Z., Qu, W., Qiao, H., Lieberman, B. P., Plössl, K., and Kung, H. F. (2012) Synthesis and evaluation of 18F labeled alanine derivatives as potential tumor imaging agents. *Nucl. Med. Biol. 39*, 933–943.

(4) Caparrós, M., Pisabarro, A. G., and de Pedro, M. A. (1992) Effect of D-amino acids on structure and synthesis of peptidoglycan in Escherichia coli. *J. Bacteriol. 174*, 5549–5559.

(5) Stewart, M. N., Parker, M. F. L., Jivan, S., Luu, J. M., Huynh, T. L., Schulte, B., Seo, Y., Blecha, J. E., Villanueva-Meyer, J. E., Flavell, R. R., VanBrocklin, H. F., Ohliger, M. A., Rosenberg, O., and Wilson, D. M. (2020) High Enantiomeric Excess In-Loop Synthesis of d-[methyl-11C]Methionine for Use as a Diagnostic Positron Emission Tomography Radiotracer in Bacterial Infection. *ACS Infect. Dis. 6*, 43–49.

(6) Parker, M. F. L., Luu, J. M., Schulte, B., Huynh, T. L., Stewart, M. N., Sriram, R., Yu, M. A., Jivan, S., Turnbaugh, P. J., Flavell, R. R., Rosenberg, O. S., Ohliger, M. A., and Wilson, D. M. (2020) Sensing Living Bacteria in Vivo Using d-Alanine-Derived 11C Radiotracers. *ACS Cent. Sci. 6*, 155–165.
